# Image Edge Detection Methods in Perimeter Security Systems Using Distributed Fiber Optical Sensing

**DOI:** 10.3390/s22124573

**Published:** 2022-06-17

**Authors:** Petr Dejdar, Pavel Záviška, Soběslav Valach, Petr Münster, Tomáš Horváth

**Affiliations:** 1 Department of Telecommunications, Faculty of Electrical Engineering and Communications, Brno University of Technology, Technická 12, 61600 Brno, Czech Republic; xzavis01@vutbr.cz (P.Z.); valach@dfcdesign.cz (S.V.); munster@vut.cz (P.M.); horvath@feec.vutbr.cz (T.H.); 2 Czech Optical Solutions s.r.o., Vavrečkova 5262, 76001 Zlín, Czech Republic

**Keywords:** edge detection, intruder detection, phase-OTDR, signal-to-noise ratio, vibration sensing

## Abstract

This paper aims to evaluate detection algorithms for perimeter security systems based on phase-sensitive optical time-domain reflectometry (Φ-OTDR). Our own designed and developed sensor system was used for the measurement. The main application of the system is in the area the perimeter fencing intrusion detection. The system is unique thanks to the developed motherboard, which contains a field-programmable gate array (FPGA) that takes care of signal processing. This allows the entire system to be integrated into a 1U rack chassis. A polygon containing two different fence types and also cable laid underground in a plastic tube was used for testing. Edge detection algorithms using the Sobel and Prewitt operators are considered for post-processing. The comparison is made based on the signal-to-noise ratio (SNR) values calculated for each event. Results of algorithms based on edge detection methods are compared with the conventional differential method commonly used in Φ-OTDR systems.

## 1. Introduction

Currently, fiber optic sensing systems are very common in real operations. Initial costs decrease with the number of installations and the number of solutions increases. With the growing number of optical cable installations, new possibilities for using this medium for non-data applications are offered, such as the transmission of ultrastable quantities or sensing of acoustic/mechanical vibrations and temperature in the immediate vicinity of fibers. Optical fiber sensors (OFS) are now mainly used with new installations but in special cases, these can also be used on existing infrastructures and, in some cases, on fibers with active data transmission. This reduces the cost of the initial installation of optical routes, and thus a cheaper sensing system.

OFS can also be used in places where common electronic sensors cannot be used because of high temperature, humidity, electromagnetic interference, etc. [1]. Furthermore, due to its size, the precision of measurement, and wide range of measurement quantities, such as temperature, pressure, or vibration [1,2], it is increasingly used in non-standard conditions, such as aircraft and shuttle sensors [3]. At present, it is necessary to secure various private and public facilities, such as buildings, land, airports, or various infrastructures (water, oil, or gas pipelines) against damage or intrusion. Due to their advantages, fiber optic distributed sensing systems are gaining attractivity in this area [4,5,6,7,8].

**Figure 1:**
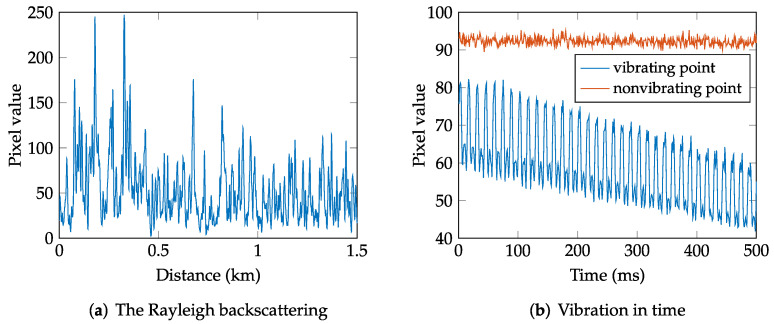
(**a**) One Rayleigh scattering response from the fiber; (**b**) time measurement showing idle state (red) and vibration (blue).

This article, which is an extended version of the conference paper [9], aims to present a distributed fiber optic sensing system based on Φ-OTDR, which has a sufficient spatial resolution, comparison of selected methods used for signal processing, and the results from real testbed measurement. A typical back-reflected signal from a fiber (one period of the reflected signal) from measurement by the Φ-OTDR system is shown in Figure 1a. Figure 1b shows a vibration in time. The red color represents the idle state, while the red-blue signal is a sinusoidal signal with a frequency of 70 Hz generated with a speaker. The entire experiment deals with vibrations caused by hitting a fence, an optical fiber box, and walking in a defined space at different distances from the fiber.

## 2. State of the Art

Fiber optic interferometers, fiber Bragg gratings (FBGs), and distributed optical sensors based on optical time-domain reflectometry (OTDR) are the most widely used in the field of optical fiber sensor systems for perimeter security. In the field of interferometers, the Michelson interferometer is used very often because of its simplicity. Authors in [10] describe perimeter security systems and signal processing algorithms for event classification. Unfortunately, it can only determine whether the perimeter has been disturbed or not, but localization is not possible. A possible solution to this problem may be the use of a zone Michelson interferometer [11], which can at least partially determine the zone where the perimeter was disturbed.

Among interferometric methods for perimeter security, it is also very advantageous to use a dual Mach–Zehnder interferometer (DMZI), which is most often used to detect cutting, and manipulation with optical fibers [12] or for the classification of falling rocks, climbing, and hitting fibers [13] and other possible typical intrusion events [14]. With DMZI, there is also the possibility of event localization [15]. The setup of this interferometer can significantly increase the cost of the entire system.

Another option to secure the perimeter is to use FBGs as presented in [16], where special fibers with Bragg gratings are used. Based on vibration type, the perimeter disturbance can be classified. These systems usually contain several FBGs in a series to approximately localize atypical events, as presented in [17,18,19].

OTDR-based sensing systems can usually determine the location of the event, and in combination with algorithms of signal processing or machine learning algorithms that are used, individual events can be classified. One option is to use a coherent optical time-domain reflectometry (C-OTDR) [20] or phase-sensitive optical time-domain reflectometry (Φ-OTDR) [21,22].

In the last decade, Φ-OTDR-based vibration sensor systems have made great progress. In addition to the improved schemes of Φ-OTDR, new signal processing methods have been designed to better detect vibrations from Rayleigh backscattering traces and improve key factors of the detection, such as the signal-to-noise ratio (SNR) and spatial resolution (SR).

Early detection techniques were based on the moving averaging and moving differential method [23,24]. To reduce the time-domain noise and improve the SNR, wavelets are very often used in Φ-OTDR systems. Quin et al. proposed the approach utilizing the continuous wavelet transform [25] and multiscale wavelet denoising [26]. Multiscale wavelet decomposition with a simple backpropagation neural network was proposed by Wu et al. in [27]. Additionally, empirical mode decomposition (EMD) was used either alone [28] or in combination with the wavelet denoising method [29]. Curvelet-based denoising for Φ-OTDR systems was presented in [30].

Statistics in the form of the signal kurtosis method are presented in [31]. A simultaneous SNR enhancement of detection of both location and frequency information using FFT was introduced by Yue et al. in [32]. Furthermore, the correlation dimension method was proposed to improve the detection of multiple locations and solve the coherent fading problem [33].

In addition, some methods locate the perturbation information from two-dimensional (2D) images composed of Rayleigh traces, such as edge detection using Sobel operator [34], Prewitt operator [35], or 2D bilateral filtering [36].

The overall progress of Φ-OTDR systems is summarized in a recent overview [37]. Table 1 shows parameters of similar systems that have been published. External AD converters have been used for systems proposed in [38,39,40], so these systems, unlike ours, are not suitable for industrial use. These systems require a PC for data processing, however, in our system is the sampling performed by an FPGA card. Another advantage of our system is that it has been designed for the use on cables on the fence, while other systems are designed for the use on buried cables. The system was therefore designed with regard to the possible stronger disturbance caused by environmental influences.

## 3. Methodology

**Figure 2:**
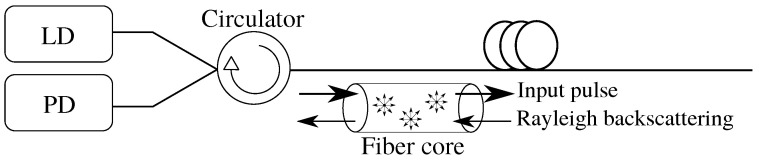
Principle scheme of OTDR.

The Rayleigh backscatter measurement, known as OTDR, has been known for decades and dates back to the 1980s [45,46,47]. In telecommunications, OTDR is commonly used to measure optical path parameters. The principle of OTDR consists of sending a high-power pulse of optical radiation generated by a laser diode (LD) into the fiber under test, where a small part of the light is back-scatter from each part of the fiber. Based on knowledge of the power level of the backscattered light from the fiber and knowledge of time, the attenuation profile of the measured fiber can be evaluated. The backscattering is captured by a photo-detector (PD), stored in time series, and averaged as shown in Figure 2.

The backscattered power $P_{R}$ can be computed as:

(1) $$P_{R}=PS\alpha_{s}W\frac{v}{2},$$

where *P* is the power of the transmitted pulse, $\alpha_{s}$ is the Rayleigh scatter loss, *W* is the width of the pulse, and *v* is the light velocity in the fiber, taken from [47] (EQ1). The parameter *S* describes the portion of the captured light [47].

The distance of the measured reflection (*l*) can then be determined using the formula:

(2) $$l=\frac{cT}{2n_{g}},$$

where *c* is the speed of light in a vacuum, *T* is the delay between sending the pulse and receiving its reflected component, and $n_{g}$ is the refractive index. The division by 2 is caused by the double distance traveled caused by the pulse time to the sensed place and the same time for receiving on the photo-detector.

Φ-OTDR is a method, most likely based on the [48] patent. Compare to basic OTDR, Φ-OTDR does not deal primarily with fiber characteristics, but with changes measurement of local phase changes.

**Figure 3:**
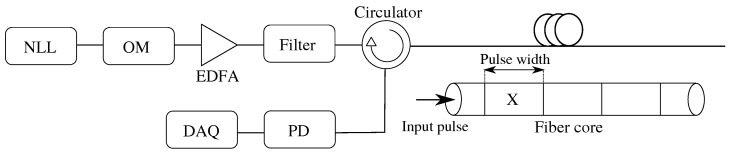
Basic scheme of Φ-OTDR.

Assuming that the internal structure of the fibers does not change, which can be assumed to be at an idle state, it is therefore possible to use Φ-OTDR to detect vibrations around the fiber. According to the review [37,49,50], it is necessary to use highly coherent radiation to generate signals, for example, using a narrow line-width laser (NLL). An optical modulator (OM) with a signal generator should be used to generate pulses. An erbium-doped fiber amplifier (EDFA) is often used as a booster to increase the power level of the pulse. EDFAs produce amplified spontaneous emission (ASE) noise, which should, if possible, be filtered out by a filter, as stated in [37]. It is also possible to use EDFA as a pre-amplifier [49] after passing through the sensing fiber, before measuring the signal using PD. Subsequently, the signal is sampled using a data acquisition (DAQ) card and finally processed by post-processing. A typical setup is shown in Figure 3.

When setting the system parameters, it is necessary to pay attention to the correct setting of the pulse period ($P_{P}$). The pulse period should be $P_{P}\geq 2L/v_{g}$, where *L* is the length of the fiber, and $v_{g}$ is the group velocity of the pulse. According to the Nycquist theorem, the maximum possible measurable frequency then corresponds to:

(3) $$f_{\max}\leq \frac{1}{2P_{P}}\leq \frac{1}{2}\left(\frac{v_{g}}{2L}\right).$$

The pulse length then affects another parameter of Φ-OTDR, which is the spatial resolution. This can be defined as:

(4) $$SR=\frac{c\tau_{p}}{2n_{g}},$$

where $\tau_{p}$ is the pulse duration. Another parameter is the maximum repetition rate, which can be defined as the number of pulses sent in 1 s. We can calculate this parameter as follows:

(5) $$f_{s,ac}=\frac{c}{2n_{g}L}.$$

The last parameter that influences the range of the system is the SNR, which is most affected by the power of the input pulse.

Evaluation of Φ-OTDR is performed by the sampling of the detected signal in the photo-detector, where the system result was achieved by subtracting every two consecutive traces and computing the absolute difference. The number of periods per second corresponds to the repetition rate. SNR can be increased with DAQ properties, where it is possible to increase the sample rate and bit-resolution parameters.

## 4. Experimental Setup

**Figure 4:**
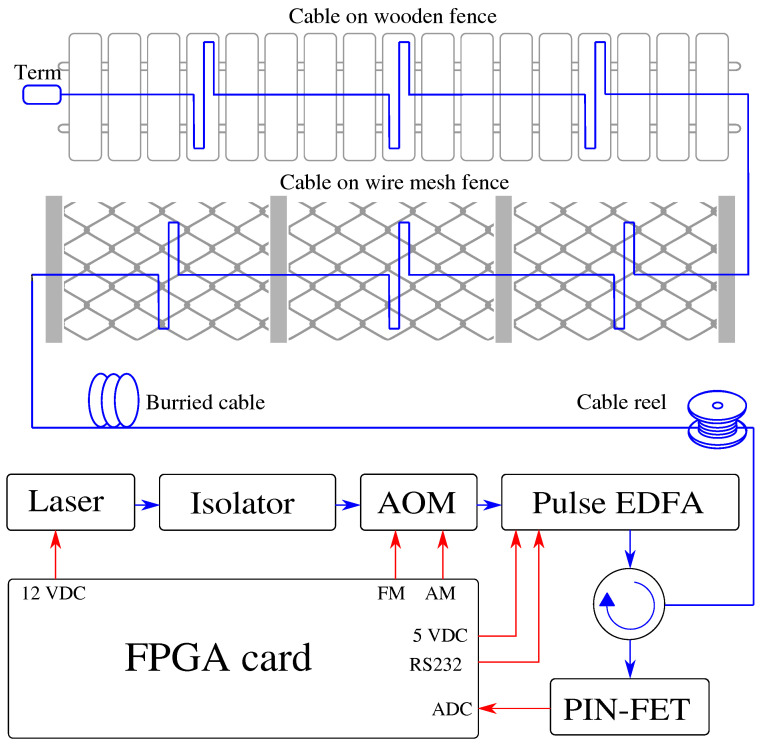
Scheme of a perimeter monitoring system based on Φ-OTDR.

The experimental setup is shown in Figure 4. The electrical part is marked by red color, and the optical part is marked by blue color. It consists of a narrow linewidth laser that generates an optical signal followed by an isolator, which protects the laser from damage when the light is reflected back to the source. An acoustic-optical modulator (AOM) is used for signal modulation. Through the circulator, pulses are transmitted to the fiber under test (FUT). An EDFA is used to amplify the power of the pulses before they enter the circulator, which directs the optical pulses to the FUT and directs the reflected signals from the fiber to the PIN-FET diode for signal processing.

The individual components are controlled by our self-designed (FPGA) card, which powers the laser, sends signals to the AOM, powers, and communicates EDFA via RS232, and contains an analog-to-digital converter (ADC) to convert the optical signal from the PIN-FET diode to electric, which is further processed.

System on a Chip (SoC) is used as the main component of the acquisition unit, which combines the Acorn RISC Machine (ARM) processor system with the Cortex A53 core and the Kintex UltraScale+ FPGA series. It is a compact solution that provides support for all the required peripherals. The card is powered by a standard ATX connector and is further supplemented with power supplies for all digital circuits in levels from 0.85 V to 3.3 V and also with power management for external components. The sampling, control, and monitoring of resources are performed using full programmable power monitor from LatticeSemi.

FPGA card also contains the direct digital synthesis (DDS) module (operating at a frequency of 1 GS/s) is controlled by a keying signal and is used to generate pulses. The serial peripheral interface (SPI) realizes the DDS setting (frequency, amplitude, and phase).

The AXISDMA unit ensures the transfer of a selected part of the data from the AD converter to the main memory of the processor and subsequently makes the data available for supervision and further analysis at a higher level. Data flow will be already reduced in the FPGA in the operational state. For more information on the FPGA card see [51].

The sensing system was set according to the parameters in Table 2 for the best SNR and optimal spatial resolution. The selected pulse length is sufficient for a test polygon that measures only 6.5 km and is described below. The pulse frequency and sampling frequency have been intentionally reduced due to memory savings and the associated huge data processing problems.

**Figure 5:**
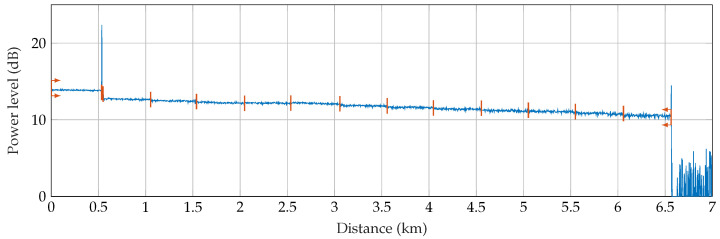
OTDR report from the testbed.

A 12-fiber outdoor optic cable was used to test the Φ-OTDR for perimeter security. The length of the cable is 500 m and the individual fibers are spliced together so that a total length of 6000 m is created. In addition to the 6 km of optical test loop, a 500 m long launch cable was used at the beginning, as can be seen in Figure 5. Subsequently, the cable rises again to the surface and is wound on a 40-m-long wire mesh fence and then on a 40-m-long wooden fence. A loop is always formed between the columns of both fences, as can be seen in Figure 4. Fibers in the cable are spliced together at both ends so it consists of 6 fibers oriented toward and 6 fibers oriented backward. In the end, a terminator is used to suppress the Fresnel reflection. A report of the OTDR route with marked splices can be seen in Figure 2. The total length of the route is 6.5 km. The FUT is ended by an optical terminator to eliminate reflection from the fiber end. The total attenuation of FUT is 2.853 dB at 1550 nm. The average attenuation is 0.436 dB/km. The average splice attenuation is 0.153 dB, where the maximum value reaches 0.210 dB.

**Figure 6:**
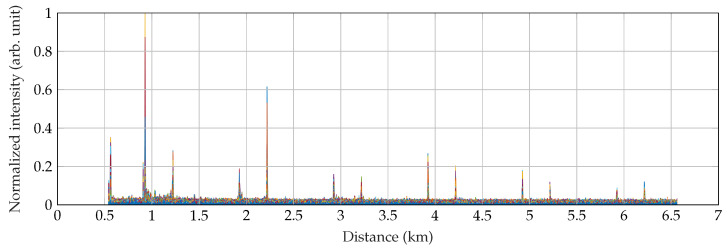
Detection of the walking can be recognized 12-times on the full length of 6 km.

Figure 6 shows the intensity of vibrations after applying the Sobel operator of size 5 × 5 (see Section 5 for a detailed description of the methods). It can be seen that the intensity of vibrations, and thus the SNR, decreases with distance, which is an effect related to the decreasing power level of the pulses. In Figure 6 we can also see six pairs of events that repeat periodically.

## 5. Methods

The goal of the signal processing methods is to detect vibrations based on the change in the Rayleigh backscattering traces. This goal can be simply achieved by subtracting every two consecutive traces and computing the absolute difference, which is usually referred to as the conventional differential (CD) method.

Formally, this process can be expressed as:

(6) $$d\left(i\right)=\left|r(i+1)-r(i)\right|,$$

where r(i) is the *i*-th of the *N* scattering traces, and d(i) is the *i*-th differential trace.

Under ideal working conditions, the conventional differential method can be efficiently used because of its high sensitivity to an intruder and very inexpensive computational cost, making it suitable for real-time detection. Nevertheless, in real conditions, the performance of the Φ-OTDR system is influenced by the limiting performance of the laser source, the finite extinction ratio of the optical modulator, thermal noise in electrical components, and the sensing fiber [37].

To cope with the negative effects of the vibration detection in real conditions and at least partially eliminate the noise, the Rayleigh backscattering traces can be preprocessed by computing the moving average [23,24], such that:

(7) $$p_{\mu }\left(j\right)=\frac{1}{\mu }\sum_{i=j}^{\mu -1+j}r\left(i\right)\;\mathrm{for}\;j=0,\ldots ,\mu -1,$$

where $p_{\mu }\left(j\right)$ denotes *j*-th processed trace obtained by computing the average of μ traces. From such processed traces, the vibrations (i.e., the changes of Rayleigh traces in time) can be detected via the CD method according to (6).

However, even though the averaging reduces the noise, it also smoothes out the vertical edges, which are important for accurate detection. Therefore, more advanced methods that do not suffer from this disadvantage have been developed to improve the signal-to-noise ratio and thus also the detection accuracy.

**Figure 7:**
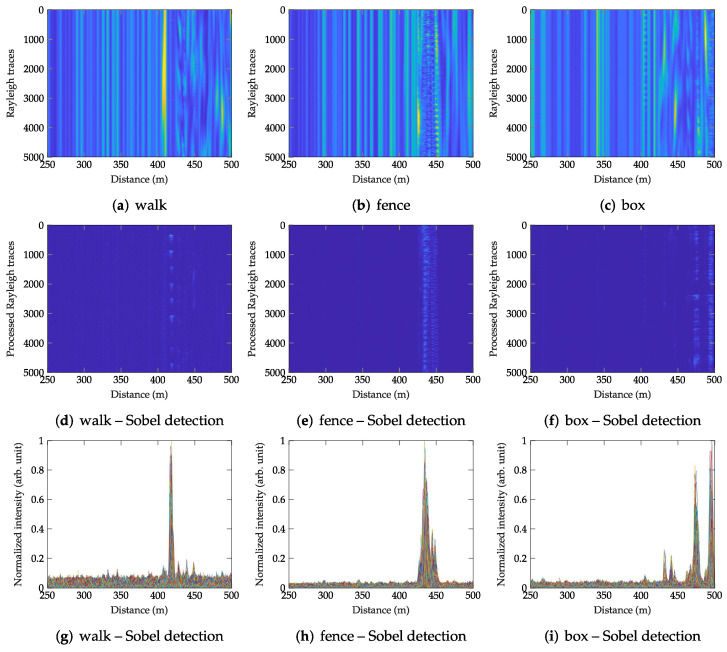
Images of consecutive Rayleigh backscattering traces for the three testing scenarios (walk, fence, box) and the respective images of location information using the Sobel edge detection method with the kernel of size 5 × 5 pixels.

Well-performing and computationally inexpensive methods are the methods based on image processing [34,35]. The image (also called the waterfall graph) can be composed of several consecutive Rayleigh backscattering traces, e.g., as shown in Figure 7a–c, where every row of the image is one backscattering trace (see Figure 1a). Since external perturbations cause significant variation in the backscattering traces (see Figure 1b), image edge detection in the vertical direction is useful to highlight the vertical edges and smooth the pseudo-edges and differences on the horizontal axis.

The edges in the vertical direction can be detected by convolving the image with the Sobel or Prewitt operator, both of which are first-order difference operators to estimate the image gradient. The definition of these operators for the vertical direction is:

$$S_{y}^{3}=\left[\begin{array}{ccc} 1 & 2 & 1\\ 0 & 0 & 0\\ -1 & -2 & -1 \end{array}\right],\;P_{y}^{3}=\left[\begin{array}{ccc} 1 & 1 & 1\\ 0 & 0 & 0\\ -1 & -1 & -1 \end{array}\right],$$

$$S_{y}^{4}=\left[\begin{array}{cccc} 2 & 5 & 5 & 2\\ 1 & 2 & 2 & 1\\ -1 & -2 & -2 & -1\\ -2 & -5 & -5 & -2 \end{array}\right],\;P_{y}^{4}=\left[\begin{array}{cccc} 3 & 3 & 3 & 3\\ 1 & 1 & 1 & 1\\ -1 & -1 & -1 & -1\\ -3 & -3 & -3 & -3 \end{array}\right],$$

$$\;S_{y}^{5}=\left[\begin{array}{ccccc} 2 & 3 & 6 & 3 & 2\\ 3 & 4 & 6 & 4 & 3\\ 0 & 0 & 0 & 0 & 0\\ -3 & -4 & -6 & -4 & -3\\ -2 & -3 & -6 & -3 & -2 \end{array}\right],\;P_{y}^{5}=\left[\begin{array}{ccccc} 2 & 2 & 2 & 2 & 2\\ 1 & 1 & 1 & 1 & 1\\ 0 & 0 & 0 & 0 & 0\\ -1 & -1 & -1 & -1 & -1\\ -2 & -2 & -2 & -2 & -2 \end{array}\right],$$

where the upper index represents operator size and the lower index denotes the direction.

The processed Rayleigh traces using the Sobel operator with the kernel size 5 × 5 are depicted in the form of waterfall graphs in Figure 7d–f. Moreover, individually processed Rayleigh traces are for illustration plotted in Figure 7g–i.

## 6. Experiments and Results

The experiments of the Φ-OTDR detection system were performed in three different scenarios. The first scenario was designed to detect outdoor walking above the cable in PVC pipe buried approx. 60 cm underground. The experiments were performed by a person of about 95 kg with a walking speed of 4.5 km/h. The walk along the fiber was realized in two ways. The first way was walking a person across the cable. The second way was a walk along the buried cable. The distance of 2 m from the buried cable is used to compare the individual methods of edge detection.

In the second and third scenarios, we simulated the perturbations by a person who knocked on the wire mesh fence and the plastic fiber optic box, where the fibers were spliced. These three scenarios are further referred to as “walk”, “fence” and “box”.

To identify the perturbations on the fiber, we compared several different methods. First, we computed the simple conventional differential method to serve as a reference. This method was also used together with the moving average method with three different lengths of the averaging filter (μ∈{5,10,20}). Finally, we applied the Sobel and Prewitt edge detection methods with three different sizes of the convolutional kernels (3 × 3, 4 × 4 and 5 × 5).

The quality of detection was measured using the signal-to-noise ratio (SNR), which is defined as the power ratio between signal (detection peak) and noise, formally:

(8) $$\mathrm{SNR}=10\log_{10}\frac{P_{\mathrm{signal}}}{P_{\mathrm{noise}}}.$$

**Figure 8:**
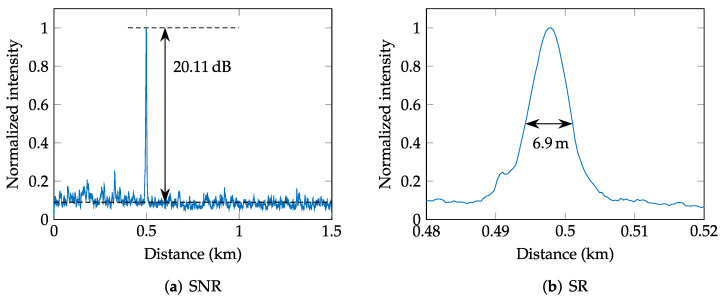
Example of SNR and SR measurements.

As presented in Figure 8a, the 12-fiber outdoor optic cable used for the detection causes 12 detected peaks of one event. However, to compute the SNR, we only exploit the first peak, which usually has the highest intensity due to the higher power level of the pulses. The level of noise $P_{\mathrm{noise}}$ is then computed as an average noise level of the intensity curve, which is taken as the upper envelope of the individual intensities (processed Rayleigh traces). The process of computing the SNR is displayed in Figure 8a.

**Figure 9:**
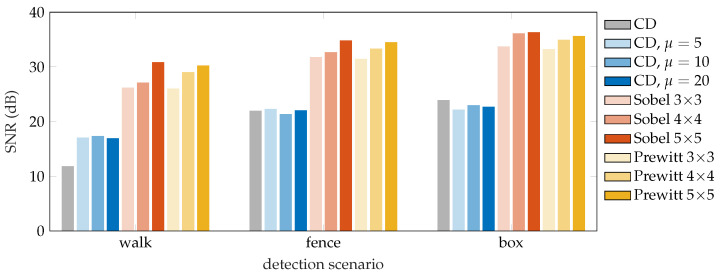
SNR results of the vibration localization for compared algorithms.

The SNR results obtained by the individual methods are displayed in Figure 9. The results show that all testing methods managed to successfully detect the vibrations in all three scenarios, nonetheless, the CD method achieved significantly worse SNR. Applying the moving average filter on the Rayleigh traces before the CD method itself seems to slightly help in cases, where the SNR obtained using the CD method is not very high (such as in the walk scenario). In this case, the improvement in SNR was approximately 5 dB. However, the averaging seems to have a negligible or even slightly negative effect on the achieved SNR in scenarios, where the perturbations were quite strong (such as knocking on the fiber box). Here, the averaging worsened the achieved SNR by approximately 1 dB.

The 2D edge detection methods proved to consistently deliver high-quality detection results in each of the testing scenarios and outperformed the conventional differential method by at least 10 dB. In general, it holds that a larger kernels produce higher SNR values, and the size of the kernel has a greater impact on the final SNR than the type of edge detection operator. Nevertheless, the Sobel operator performs marginally (less than 1 dB) better in most cases.

**Figure 10:**
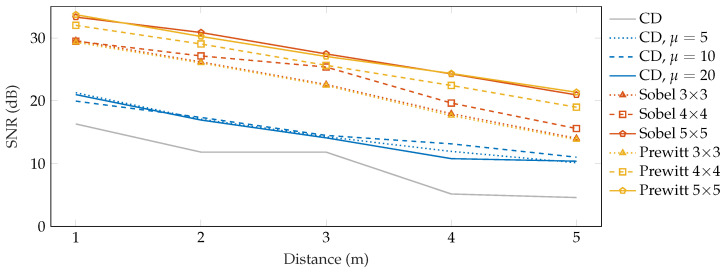
SNR results of the walk localization depending on the distance from the sensing fiber.

Apart from the overall SNR in different scenarios, we also tested the sensitivity of the proposed detection system in dependence on the perpendicular distance from the fiber. The results obtained in this experiment are shown in Figure 10 and they indicate that the achieved SNR decreases approximately by 3 dB with every meter. The plain CD method turns out to be insufficient for distances greater than 3 m with the achieved SNR of ca 5 dB since SNR below 6 dB is considered bad and may result in false-negative detections. As noted in the previous experiment, averaging helped to improve the SNR by ca 5 dB in the walk scenario. However, 2D edge detection methods with operator size 5 × 5 achieved an average SNR above 21 dB at a distance of 5 m, suggesting that this system is capable of reliably detecting an intruder even further from the fiber.

Along with the SNR, the second parameter that can be evaluated from the intensity function is the real spatial resolution (SR), which is determined as the width of the intensity peak in the middle of the peak (see Figure 8b for illustration). Nevertheless, the real spatial resolution depends more on the parameters of the Φ-OTDR system (pulse length, vibration intensity, photo-detector bandwidth) than on the signal processing itself, and it is not as important as the achieved SNR. The average achieved spatial resolution was 8.8 m for the walk scenario, 26 m for the knocking on the fiber box event, and finally 40 m for the wire mesh fence, which corresponds to the entire length of the fence.

Different spatial resolution values for individual testing scenarios are caused mainly by the different lengths of the vibrating sections. For example, knocking on the wire mesh fence causes vibrations on the whole fence, which is naturally reflected in the collected backscattering traces, compared to the scenario of the walk, where the individual steps cause vibrations only on a small segment of the fiber. Thus, a better spatial resolution could be achieved, for example, by better attachment of the fiber to the fence or by separating the individual sections of the fence.

## 7. Conclusions

A perimeter security detection system based on Φ-OTDR and completely controlled by FPGA was designed and implemented. This system was tested on a prepared test path, which contained a typical location of optical security cables, such as buried in the ground or mounted on a secure fence. Testing acquired enough data from various events that commonly occur when disrupting the perimeter.

A total of three detection methods were used to detect the disruptions—Sobel and Prewitt edge detection methods with three different kernel sizes, and a simple conventional differential method for reference. We also exploited the moving average method to reduce the noise level from the obtained Rayleigh traces. To quantify the quality of vibration detection, we computed the SNR. From the obtained results, it is possible to say that the best SNR was achieved using the Sobel operator of size 5 × 5. Furthermore, an experiment was performed to determine the dependence of the walking distance from the fiber on the SNR. It was shown that the SNR decreases linearly with the distance from the fiber by approximately 3 dB. Unlike most of the research on vibration detection in Φ-OTDR, our experiments were carried out on a real route with real examples of perimeter disturbances.

In future work, the system should be tested on a real testbed for a long time. Based on a larger number of measurements, the accuracy of the entire system should then be evaluated. Subsequently, progress should be made to automatically detect events and evaluate the accuracy of the system, and, if necessary, evaluate the false alarm percentage. The system is currently being serviced, and therefore all event evaluations are performed by the operator.

## Figures and Tables

**Table 1:** Comparison of the Φ-OTDR system.

Φ-OTDR	Repetition Rate	Sampling Rate	Laser Line-Width	Laser Power
Ying [41]	50 kHz	100 MS/s	5 kHz	10 dBm
Franciscangelis [38]	20 kHz	2500 MS/s	200 kHz	0 dBm
Gang [42]	1 kHz	–	5 kHz	13 dBm
Tomboza [43]	–	100 MS/s	0.075 kHz	11 dBm
Filograno [44]	–	200 MS/s	–	13 dBm
Jason [39]	20.2 kHz	1000 MS/s	0.1 kHz	16 dBm
Daisuke [40]	10 kHz	1250 MS/s	1 kHz	–
Our system	upto 20 kHz	250 MS/s	<0.1 kHz	15 dBm

**Table 2:** Parameters of the Φ-OTDR system.

pulse width	50 ns
sampling frequency	250 MHz
repetition rate	1000 Hz

## Data Availability

Not applicable.
